# Decision aid on radioactive iodine treatment for early stage papillary thyroid cancer - a randomized controlled trial

**DOI:** 10.1186/1745-6215-11-81

**Published:** 2010-07-26

**Authors:** Anna M Sawka, Sharon Straus, James D Brierley, Richard W Tsang, Lorne Rotstein, Gary Rodin, Amiram Gafni, Shereen Ezzat, Lehana Thabane, Kevin E Thorpe, David P Goldstein

**Affiliations:** 1 Division of Endocrinology, Department of Medicine, University Health Network and University of Toronto, Toronto, Ontario, Canada; 2 Department of Medicine, St. Michael's Hospital and University of Toronto, Toronto, Ontario, Canada; 3 Department of Radiation Oncology, University Health Network and University of Toronto, Toronto, Ontario, Canada; 4 Department of Surgery, University Health Network and University of Toronto, Toronto, Ontario, Canada; 5 Department of Psychosocial Oncology, University Health Network and University of Toronto, Toronto, Ontario, Canada; 6 Department of Clinical Epidemiology and Biostatistics, McMaster University, Hamilton, Ontario, Canada; 7 Department of Biostatistics, Keenan Research Centre, Li Ka Shing Knowledge Institute of St. Michael's Hospital and the Dalla Lana School of Public Health, University of Toronto, Toronto, Ontario, Canada; 8 Department of Otolaryngology, University Health Network and University of Toronto, Toronto, Ontario, Canada

## Abstract

**Background:**

Patients with early stage papillary thyroid carcinoma (PTC), are faced with the decision to either to accept or reject adjuvant radioactive iodine (RAI) treatment after thryroidectomy. This decision is often difficult because of conflicting reports of RAI treatment benefit and medical evidence uncertainty due to the lack of long-term randomized controlled trials.

**Methods:**

We report the protocol for a parallel, 2-arm, randomized trial comparing an intervention group exposed to a computerized decision aid (DA) relative to a control group receiving usual care. The DA explains the options of adjuvant radioactive iodine or no adjuvant radioactive iodine, as well as associated potential benefits, risks, and follow-up implications. Potentially eligible adult PTC patient participants will include: English-speaking individuals who have had recent thyroidectomy, and whose primary tumor was 1 to 4 cm in diameter, with no known metastases to lymph nodes or distant sites, with no other worrisome features, and who have not received RAI treatment for thyroid cancer. We will measure the effect of the DA on the following patient outcomes: a) knowledge about PTC and RAI treatment, b) decisional conflict, c) decisional regret, d) client satisfaction with information received about RAI treatment, and e) the final decision to accept or reject adjuvant RAI treatment and rationale.

**Discussion:**

This trial will provide evidence of feasibility and efficacy of the use of a computerized DA in explaining complex issues relating to decision making about adjuvant RAI treatment in early stage PTC.

**Trial registration:**

Clinical Trials.gov Identifier: NCT01083550

## Background

Thyroid cancer is increasingly being diagnosed in North America and Europe [1-5]. Papillary thyroid carcinoma (PTC) accounts for the majority of these newly diagnosed cases [1,6]. Most patients diagnosed with PTC have early stage disease and are at low risk of dying from thyroid cancer [7]. It is currently recommended that most patients with PTC undergo surgical removal of the thyroid (total or near-total thyroidectomy), to be followed by life-long thyroid hormone therapy [8-10]. Adjuvant radioactive iodine (RAI) treatment or the option of no adjuvant RAI treatment (with close medical follow-up) may be offered in early stage PTC after thyroidectomy [8,11]. There are no long-term randomized controlled trials examining whether adjuvant RAI treatment reduces the risk of thyroid cancer-related recurrence or mortality in early stage PTC, and the best available observational evidence includes conflicting reports of treatment benefit [11]. The potential risks, benefits, reproductive considerations, and implications for disease follow-up must be considered in RAI decision making. However, in early stage PTC, adjuvant RAI treatment decision-making is complex and subject to some uncertainty [11].

In a recent focus group study conducted in Canada [12], thyroid cancer survivors reported receiving conflicting recommendations on adjuvant post-surgical RAI treatment from healthcare providers and insufficient information on rationale, risks, and uncertainties relating to this treatment. In many cases, no choice was offered and patients felt the treatment was imposed on them. This lack of clarity in communication has led thyroid cancer survivors to call for the development of plain-language, evidence-based information on this topic, such as in a DA [12]. Decision aids are instruments used to inform patients about available treatment options which have been found to generally improve general patient knowledge, to result in more realistic patient treatment expectations, to increase active patient participation in decision making, and to reduce indecisiveness, when compared to usual care; DA's may be computerized or paper-based or a combination of both [13].

Our aim in this randomized controlled trial, is to evaluate, in individuals with early stage PTC who have had thyroidectomy, the effect of a computer-based DA on the following outcomes: a) knowledge about PTC and RAI treatment (primary outcome), b) decisional conflict, c) decisional regret, d) client satisfaction with information received about RAI treatment, and e) the final decision to accept or reject adjuvant RAI treatment and the rationale for this decision. The comparator group will be individuals with early stage PTC who have not been exposed to the DA but who have received usual care from their physicians. We present herein the protocol that we are using to conduct this trial.

## Methods

### Design

In order to evaluate this DA, we propose to conduct a single-centre, patient-level randomized controlled trial, comparing exposure to the DA (with usual care) to a control group of usual care. The study protocol has been approved by the University Health Network Research Ethics Board. Signed informed consent will be obtained from all participants.

### Setting

Administration and testing of the DA will be conducted in a research office at the Toronto General Hospital site of the University Health Network in Toronto, Canada. The University Health Network is a tertiary care referral centre for thyroid cancer care. Participants not cared for at the University Health Network will be eligible for participation in the trial, providing the report of their thyroid surgical pathology may be reviewed by investigators, to ascertain eligibility.

### Participants

#### Inclusion criteria

Participants in the study will include individuals with early stage papillary carcinoma in whom either accepting or rejecting adjuvant RAI treatment are reasonable, given the lack of clear-cut evidence guiding therapy. Potentially eligible patient participants will include: individuals who have had complete resection of their thyroid at surgery (total or near-total thyroidectomy, or hemi- [subtotal] with completion thyroidectomy) at the age of 18 years or older, in the time since September 1, 2009 (last thyroid surgery on or after September 1, 2009). The papillary thyroid cancer must have been of TNM pathologic stage pT1 or pT2, N0 (or Nx), M0 (or Mx) (TNM stage, American Joint Committee on Cancer VI [14]) (ie. primary tumor size 1-4 cm, no known positive lymph nodes at the time of primary surgery, no extension of the tumor outside the thyroid, no venous or lymphatic invasion, and no known distant metastases at primary surgery, with no tall cell features, as per surgical pathology report). Individuals concurrently diagnosed with medullary or anaplastic or poorly differentiated thyroid cancer or thyroid lymphoma are not eligible for the study. Participants must be able to communicate in spoken and written English and able to use a computer and provide informed consent on their own (without any need for translation). The reason for the English language fluency criterion is that the DA is currently available only in English.

#### Exclusion criteria

Individuals who have already received RAI treatment for thyroid cancer are not eligible for this study, since the decision to accept RAI treatment would have already been made in such individuals. However, individuals meeting inclusion criteria who are on the waiting list for RAI treatment may be eligible, since their decision could still be subject to change. Participants not meeting the inclusion criteria will be ineligible. Individuals who have been temporarily stopped taking thyroid hormone (for the purpose for testing or RAI treatment), will not be eligible for the study while off this medication, since it could potentially impact cognitive ability. Individuals who are unwilling for investigators to confirm their pathologic stage of thyroid cancer through review of pathology reports, will be ineligible for the study.

#### Participant recruitment

Participants will be recruited through the following means: a) review of recent pathology records reviewed at University Health Network, with mailing of information about the study to potentially eligible participants, b) review of the radioactive iodine treatment wait list at the University Health Network, with mailing of the information about the study to potentially eligible participants, c) use of posters and flyers about the study in University Health Network (hallways, patient wait areas, and clinics), d) dissemination of information about the study through internet media and possibly newsletters of a Canadian thyroid cancer support group (Thyroid Cancer Canada, formerly known as Thry'vors), e) and distribution of information about the study via e-mail (and pamphlets, if requested) to thyroid cancer physicians in the Greater Toronto Area. Recruitment materials will indicate that potentially interested participants should telephone the research office for more information. A research staff member will then inform potential participants about the trial and collect information on eligibility. All pathology reports of potential participants will be reviewed for eligibility by either the primary investigator (AMS) or co-investigator (DPG). A consent form for release of medical information will be signed by potentially eligible participants to obtain thyroid cancer surgical pathology reports from treating physicians, if this information is not already available to investigators nor provided by patients at recruitment. Patient participants will be compensated with $100 after completion of the first study visit (randomization and testing) and with an additional $100 for completion of the telephone follow-up.

### Intervention

#### Decision aid

A multi-disciplinary team of thyroid cancer physicians and methodologists were involved in development of the computerized DA. The content of the DA was based on the information needs identified by thyroid cancer survivors in a focus group study [12] and the physician members involved in development. Multiple systematic reviews of the literature were conducted in order to ensure the DA content was evidence-based [11,15-17]. The DA was subjected to pre-testing by thyroid cancer physicians, thyroid cancer survivors, and lay individuals (usability testing followed by revisions).

For participants randomized to the intervention group, the DA website will be opened for the participant by staff and the participant will be asked to self-navigate the website. The number of computer mouse clicks used to retrieve information and the duration of DA examination will be recorded for each participant in the intervention group. It will be suggested to the participant that he or she may spend up to 60 minutes reviewing the website, but he or she may finish early if desired. The participant will review the information in the DA on his or her own during one visit at the Toronto General Hospital, with no access to the website nor print-outs allowed at home. A research assistant will be present in the same room during the study visit, in case the participant has any questions about navigation of the DA. All participants in the intervention group will receive usual care from their treating physicians.

### Usual care control group

The usual care control group will be provided counselling and care from their own treating physicians, and not provided any exposure to the DA during the trial.

### Randomization

Central computerized randomization will be performed in a 1:1 ratio (variable block sizes) at a patient participant level. The randomization will be to the intervention group (exposure to the computerized DA in addition to usual care) or control group (no exposure to the DA, only usual care). The randomization will be achieved through the DA program, after the study staff enters the participant's unique identifier number, to be immediately followed by testing. Allocation will be concealed until the this testing visit. Thus, prior to the testing visit, neither the participant, study staff, investigators, nor treating physicians will be aware of the allocation.

### Outcomes, follow-up, and data collection

Participant involvement in the trial will include one visit to the Toronto General Hospital, at which point, signed informed consent for participation in the trial will be obtained, and baseline data will be collected on demographic characteristics and psychological coping style [18]). The randomization will be performed, and the intervention administered at this time (for those randomized to the DA group), and self-administered questionnaires will be completed. A telephone follow-up interview will be performed at 6 months (with telephone follow-up in subsequent months, after the decision on RAI treatment has been finalized).

To assess efficacy of the DA intervention, the primary outcome measured in this trial will be the score (number of correct responses out of 10 questions) on a self-administered written questionnaire examining knowledge about PTC and RAI treatment, which will be administered at the randomization visit (immediately after exposure to the DA in the intervention group, or after revealing no exposure to the DA in the control group). After the knowledge questionnaire is completed, decisional conflict [19,20] relating to the decision to accept or reject adjuvant RAI treatment will be assessed as a secondary outcome, using a self-administered questionnaire in all participants.

Telephone follow-up will be performed six months after the randomization visit for all participants. If, the final decision on RAI treatment has not been finalized by this time (ie. decision not yet made, or if the participant is on a waiting list for RAI treatment but has not yet received it), another telephone follow-up will be set up within the next several months (depending on timing of any planned RAI treatment or finalization of the decision). The following data will be collected by telephone interview 6 months after randomization: a follow-up interview on RAI decision-making (inquiring about the final decision about RAI treatment and rationale), a client satisfaction questionnaire on information received relating to RAI decision-making (modified from general questionnaire on client satisfaction [Miller Behavioral Style Scale, MBSS, [21]]), and a decision regret questionnaire [22,23]. If, at the 6-month follow-up phone call, the final decision on RAI treatment has not been finalized, the decisional regret questionnaire will be deferred until another telephone call to be set up in subsequent months (after finalization of the treatment decision). If a second telephone follow-up call is required, the interview questions and questionnaires administered at 6 months will be repeated.

### Statistical considerations

#### Quantitative analysis

For descriptive analyses, categorical data will be expressed as number and proportion (or percentage), whereas continuous data will be expressed as mean and standard deviation (or median and range). Independent sample t-tests will be used to compare questionnaire scores, using an intention-to-treat principle. A chi-squared analysis will be used to compare the proportion of individuals accepting RAI treatment in the intervention group compared to the control group (separate analyses for all participants, as well as excluding those on the radioactive iodine treatment wait list at the time of recruitment). Alpha = 0.05 will be used as the cut-off for statistical significance for all analyses. Missing data for any questionnaire subscales will be imputed from the mean of the rest of the questions within that subscale, for questionnaires in which subscales are established.

#### Qualitative analysis

Content analysis of the qualitative data collected in the telephone follow-up (including the decision to accept or reject RAI treatment and rationale), will be completed manually, using the notes of the telephone interviewer. The major themes will be identified using grounded theory [24-26]. Descriptive frequency analysis of coded data will also be quantified using mixed methods [27-29].

#### Sample size calculations

The sample size is calculated to detect a one point difference in the mean score of the knowledge questionnaire (relating to PTC and RAI treatment), between the intervention and usual care groups, assuming a standard deviation of 1.5 (Power and Precision software, Biostat, Inc., Englewood, NJ, USA). The intended sample size is 37 individuals per group (total of 74) (0.05 2-tailed alpha, power 0.808).

### Additional exploratory analyses

We will examine the following Spearman correlation coefficients for the baseline monitoring and blunting coping questionnaires scores (as measured by the MBSS) relative to the following: a) number of computer mouse clicks used by individuals in the intervention group to retrieve information within the DA, b) knowledge questionnaire score, c) and participant satisfaction with information received, as assessed at telephone follow-up. We will also compare the MBSS scores in individuals who ultimately receive adjuvant RAI treatment compared to those who reject it (Student's t-tests).

### Invitation for feedback from treating physicians

In the months before study completion, we will mail out a self-administered written satisfaction survey (modified from general questionnaire on client satisfaction [20]) to thyroid physicians and surgeons in active practice at the University Health Network (where the majority of patient participants are expected to be recruited). This survey will invite physicians' feedback on their experience with patients who may have been exposed to the DA intervention in the trial.

### Knowledge translation plan

After completing testing of the proposed DA and publication of the results of this study, it is planned for the decision aid to be made publicly available on the website of the University Health Network Patient Education Network website, such that it can be made available to PTC patients and treating physicians at UHN, other academic centres, and the community. We will also be sharing our findings with a Canadian thyroid cancer support group (Canadian Thyroid Cancer Canada, formerly known as Thry'vors), and sharing links to the DA website if the group is interested.

## Discussion

We have presented the design of a randomized controlled trial to evaluate the effect of a decision aid, explaining the options of adjuvant RAI treatment or no adjuvant RAI treatment, for individuals with early stage PTC. In particular we are interested in determining whether the DA may improve patient knowledge about PTC and RAI treatment, reduce decisional conflict and decisional regret, and influence treatment choice or rationale, as compared to usual care. The DA in question is unique in that it explains in detail, the current uncertainty relating to potential treatment benefits of adjuvant RAI treatment in early stage PTC.

## Abbreviations

PTC: (papillary thyroid cancer); RAI: (radioactive iodine treatment); DA: (decision aid).

## Competing interests

The authors declare that they have no competing interests.

## Authors' contributions

AMS participated in conception and design of the study, secured funding for the project, performed sample size calculations, and drafted the manuscript. SS has participated in conception and design of the study and revised the manuscript for important intellectual content. JDB, RWT, LR, SE and LT provided input in study design and revised the manuscript for important intellectual content. KET provided input in design of the study, independently checked the sample size calculations and revised the manuscript for important intellectual content. DPG participated in conception and design of the study, assisted in securing funding for the project and revised the manuscript for important intellectual content. All authors read and approved the final manuscript.

**Figure 1:**
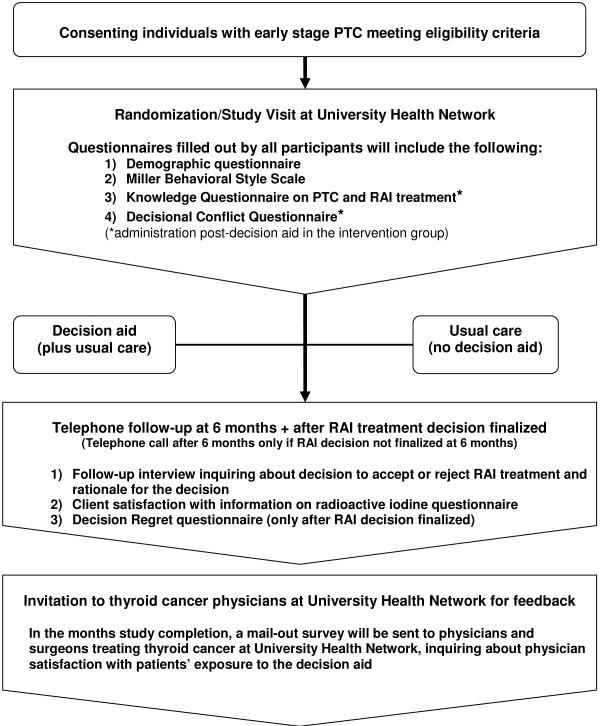
**Overview of the study**. PTC: papillary thyroid cancer, RAI: radioactive iodine.
